# The role that USAID and other development funders play in furthering mental health research

**DOI:** 10.1017/gmh.2024.105

**Published:** 2024-10-17

**Authors:** Lyla Schwartz, USAID Inclusive Development Hub and Victims of Torture Fund

**Affiliations:** USAID - Inclusive Development Hub - Victims of Torture, Washington, DC, USA

**Keywords:** Global mental health, Culture, implementation science research

## Abstract

Feb. 19, 2024.

Feb. 19, 2024.

Updated: Advancing Mental Health Research Through Collaboration.

Mental health experiences impact the well-being and functioning of populations worldwide. Over the past decade, progress has been made in documenting the prevalence of these conditions globally in both development and humanitarian contexts. Implementation science promotes the integration of evidence-based practices into real-world settings, aiming to improve outcomes by understanding and addressing implementation barriers. It bridges the gap between research and practical application in fields like healthcare, education and social services. Despite these advancements, substantial gaps in implementation research hinder the expansion of mental health policies, programs and practices. The primary objective of this brief is to discuss gaps in global mental health research and propose recommendations on how policies and programs can address critical research needs within the realm of international development and humanitarian assistance.

In addition to financial support, development funders can catalyze innovative mental health research by collaborating with research institutions, positioning themselves as champions of evidence-informed practices that resonate across diverse populations, including those who are marginalized or who are in vulnerable situations.

A particularly critical research gap exists on the efficacy and effectiveness of locally developed and/or adapted mental health services and interventions within low- and middle-income countries (LMICs) and humanitarian settings. Evidence-based programs developed in one region may not be culturally or contextually appropriate or effective elsewhere and may even prove to be harmful. Implementation science research in global mental health is limited, with existing evidence focusing on the early stages of implementation, raising concerns regarding long-term sustainability. Improved quality of research in LMICs is needed to understand cultural and contextual nuances influencing the efficacy of mental health treatment approaches. This cannot be adequately addressed without tackling the challenges researchers in the Global South often experience participating in the global research ecosystem, including obtaining funding, publishing in influential journals and accessing influential networks.

To address these challenges and bridge existing gaps in mental health research, several recommendations are proposed: First and foremost, it is crucial to support locally led research efforts in general and as a key component of addressing the recommendations below. Researchers in the Global South are best positioned to address the unique challenges (including the impacts of and approaches to addressing stigma) and needs of their communities and drive effective participatory and inclusive research that can lead to more effective, culturally appropriate interventions. They are also best positioned to identify excluded and/or underserved populations with unique needs and safely engage group members in developing and researching approaches. Additionally, the development of reliable and valid measurement instruments and methods is essential. Standardized tools for assessing mental health outcomes and intervention effectiveness can ensure consistency and comparability across different research settings; heretofore and related to the first recommendation, these have been developed based on models from the Global North that have a biomedical basis that does not resonate in many cultures and has been widely critiqued by people with mental health conditions, service users and others. It is therefore critical to ensure that new tools and resources are developed in full partnership with researchers and other stakeholders in the Global South. These ideas must work for different communities with varied views on mental health causes, symptoms, support types and related concepts.

Significant gaps in research on effective approaches for mental health promotion and prevention initiatives need to be addressed with a focus on early intervention and prevention strategies to reduce the impact of mental health symptoms and conditions on individuals and communities. A substantial research base exists identifying influential social determinants of mental health and offering a strong basis to inform additional research on prevention and early intervention, but should be combined with participatory research practices at all stages to increase impact. Development and humanitarian assistance organizations already do significant work to address some of the most influential determinants of mental health as these are often targets of the sustainable development goals (SDGs; e.g., poverty, economic consideration, inequality, violence [including gender-based violence], conflict stabilization, physical health, climate and more);research often does not clearly demonstrate which methods are most effective in addressing these challenges while also supporting mental health. However, studies do indicate that these challenges and mental health frequently influence each other, indicating that enhancing mental well-being can significantly contribute to resolving these gaps. For example, integrating mental health into HIV programs can result in significant improvements in the impact of these programs (Collins et al., 2021). In spite of increased strong evidence in this area, there are significant gaps in research that could inform decision making about which specific approaches to integrating mental health into programs addressing the SDGs and other development and humanitarian goals has the most potential for improving the results of these programs.

Funders, governments and other organizations often aspire to support initiatives that can benefit the maximum number of people within the available funds. This can only be feasibly and sustainably achieved at scale by incorporating population and systems-level approaches, including policy, regulatory and parity programs (e.g., more equitable funding for mental health services, inclusion in insurances). However, despite the promise these efforts hold, the evidence base for such approaches is limited. Expanding this foundation entails research that targets systems and population level interventions and outcomes and may include exploring influential case studies, conducting comparative analyses, assessing stakeholder engagement, evaluating scalability and systems integration and investigating the effectiveness and cost implications of mental health investments. Such efforts are critical for effectively scaling the impact of population and systems level approaches.

Research should prioritize investigating the efficacy of mental health interventions across diverse settings, including examining scalability and sustainability beyond controlled research environments. Implementation science offers useful guidelines, but participatory, locally led engagement could help develop research programs that better fit local needs, integrate other approaches (e.g., Indigenous peoples) and thus inform and develop more robust global evidence bases.

Furthermore, research on best implementation and dissemination practices are critical for ensuring that evidence-based practices are effectively translated into policy and practice. Studying implementation and dissemination processes can identify facilitators and barriers, including the often insidious impact of stigma and discrimination, to successful program delivery and uptake.

Ethical considerations for mental health research in humanitarian settings must be more fully addressed, including ensuring culturally appropriate informed consent, protecting the rights and dignity of research participants and minimizing potential harm. Moreover, prioritizing the inclusion of diverse populations in mental health research efforts (considering factors such as age, gender, ethnicity, socioeconomic status and cultural background in study design and implementation) is essential. Advancements in dissemination and implementation science are needed to facilitate the translation of research findings into practice, maximizing the impact of mental health research on policy and practice. The utilization of robust and reproducible research methods is imperative, especially considering funding challenges in many LMICs and unstable humanitarian settings, which can impact research quality. Interdisciplinary teams and collaboration among diverse stakeholders, including communities, healthcare providers, policymakers, service users and development organizations, are essential for making a meaningful impact on mental health programming incorporating research-informed practices. National actors such as ministries of health, mental health professionals and other key stakeholders actively involved in supporting mental health research and the implementation of evidence-informed practices in many LMIC settings is crucial to making sure research is responsive to local needs, constraints and priorities.

An example of research application in the development and implementation of programs is the Ensuring Quality in Psychosocial and Mental Health Services (EQUIP) initiative, a collaboration among USAID, the World Health Organization and George Washington University (Kohrt et al., 2020). EQUIP is a freely available resource for standardized approaches to assess competencies and conduct competency-based training and supervision that educational institutions, government agencies and nongovernmental organizations (NGOs) across sectors in both development and humanitarian settings can use to benchmark skills for safe and effective care. The EQUIP materials and digital platform (see www.equipcompetency.org) were developed in collaboration with NGOs working in a number of different LMICs to ensure that they were cross culturally relevant and had the scope to be adapted where necessary. Research on the use of these tools in training and supervision practices has shown an increase in helpful behaviors and reduction in potentially harmful behaviors among nonspecialist helpers (Jordans et al., 2022). Having a research-developed tool to measure these factors is critically important for increasing the quality of mental health services and, because the quality of helping relationships is strongly linked to their effectiveness, for facilitating research aimed at understanding the key components of interventions. The EQUIP initiative continues to support actors to integrate the tools and resources into their existing training and supervision practices to ensure that helpers provide safe, high-quality psychosocial support and mental health care.

In conclusion, collaborative efforts between development funders, researchers and local stakeholders can address existing gaps in mental health research and work toward more effective and equitable mental health policies and practices globally. Prioritizing locally based research, promoting prevention and early intervention and ensuring the inclusion of diverse populations can improve mental health outcomes and promote well-being for all individuals and communities.
