# Supplementary material for: The role that USAID and other development funders play in furthering mental health research
Source: Glob Ment Health (Camb). 2024 Oct 17;11:e82. doi: 10.1017/gmh.2024.105 (PMC11504921; doi:10.1017/gmh.2024.105)
Supplement: Peer review history

# Editor report: Recommendation: The role that USAID and other development funders play in furthering mental health research — R0/PR2

**DOI:** 10.1017/gmh.2024.105.pr2

**Affiliations:** Johns Hopkins University Bloomberg School of Public Health, United States

No accompanying comment.

# Editor report: Decision: The role that USAID and other development funders play in furthering mental health research — R0/PR3

**DOI:** 10.1017/gmh.2024.105.pr3

**Affiliations:** Johns Hopkins University Bloomberg School of Public Health, United States

No accompanying comment.

# Editor report: Recommendation: The role that USAID and other development funders play in furthering mental health research — R1/PR5

**DOI:** 10.1017/gmh.2024.105.pr5

**Affiliations:** Johns Hopkins University Bloomberg School of Public Health, United States

The comments in peer review have been acknowledged but the decision has been made to publish as the paper stands without further revisions being invited to address peer review comments. This is a non research content article and level of post peer review editing lies with the Editor in Chief.

# Editor report: Decision: The role that USAID and other development funders play in furthering mental health research — R1/PR6

**DOI:** 10.1017/gmh.2024.105.pr6

**Affiliations:** Johns Hopkins University Bloomberg School of Public Health, United States

No accompanying comment.
